# Post-traumatic thrombotic microangiopathy following pelvic fracture treated with transcatheter arterial embolization: a case report

**DOI:** 10.1186/s13256-018-1757-1

**Published:** 2018-08-09

**Authors:** Kaori Ikegami, Takuma Yamagishi, Junya Tajima, Yukinori Inoue, Ken Kumagai, Yasuo Hirose, Daisuke Kondo, Koji Nikkuni

**Affiliations:** 1 The Department of Emergency and Critical Care Medicine, Niigata City General Hospital, 463-7 Shumoku, Chuo-ku, Niigata, Niigata 950-1197 Japan; 2 The Department of Intensive Care Medicine, Yokohama City Minato Red Cross Hospital, 3-12-1 Shin-yamashita Naka-Ku, Yokohama, Kanagawa 231-8682 Japan

**Keywords:** TMA, Hemolysis, Thrombocytopenia, ADAMTS13, Trauma

## Abstract

**Background:**

Thrombotic microangiopathy is caused by various conditions, but few cases secondary to trauma have been reported. We present the rare case of a patient with thrombotic microangiopathy-induced high-impact trauma with hemorrhagic shock.

**Case presentation:**

An 86-year-old Japanese woman was transferred to our hospital after a traffic accident. A whole-body computed tomography scan revealed pelvic fractures with massive extravasation. She received a blood transfusion and emergency angiographic embolization. On post-traumatic day 1, she showed unexplained severe hemolysis, thrombocytopenia, and renal failure despite her stable condition. Disseminated intravascular coagulation was excluded because her activated partial thromboplastin time and prothrombin time-international normalized ratio were normal. Her fragmented red blood cell concentration was 28.8%. We suspected clinical thrombotic thrombocytopenic purpura and started plasma exchange. She recovered fully after the plasma exchange and was discharged on day 31. We eventually diagnosed thrombotic microangiopathy because her ADAMTS13 activity was not reduced.

**Conclusions:**

It is important to recognize the possibility that thrombotic microangiopathy may occur after severe trauma. In the critical care setting, unexplained thrombocytopenia and hemolytic anemia should be investigated to eliminate the possibility of thrombotic microangiopathy. Early plasma exchange may help to prevent unfortunate outcomes in patients with thrombotic microangiopathy following trauma.

## Background

Thrombotic thrombocytopenic purpura (TTP) is a life-threatening condition and medical emergency. It is characterized by a pentad of thrombocytopenia, microangiopathic hemolytic anemia, central neurological disorders, renal failure, and fever. The ADAMTS13 (also known as von Willebrand factor-cleaving protease) activity or ADAMTS antibody level is now used in the diagnosis of TTP. Plasma exchange is essential in the treatment of TTP.

Postoperative TTP has been frequently described, whereas post-traumatic TTP is extremely rare. Here we report a case of post-traumatic thrombotic microangiopathy (TMA) that was successfully treated with plasma exchange.

## Case presentation

**Fig. 1:**
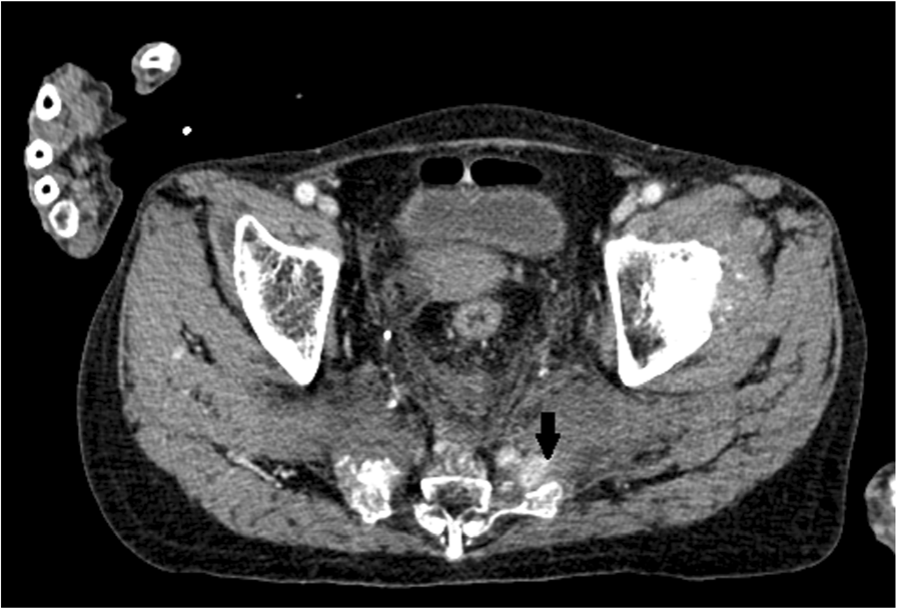
Contrast-enhanced computed tomography of the patient with pelvic fractures after trauma. The *arrow* indicates a hematoma with extravasation

**Fig. 2:**
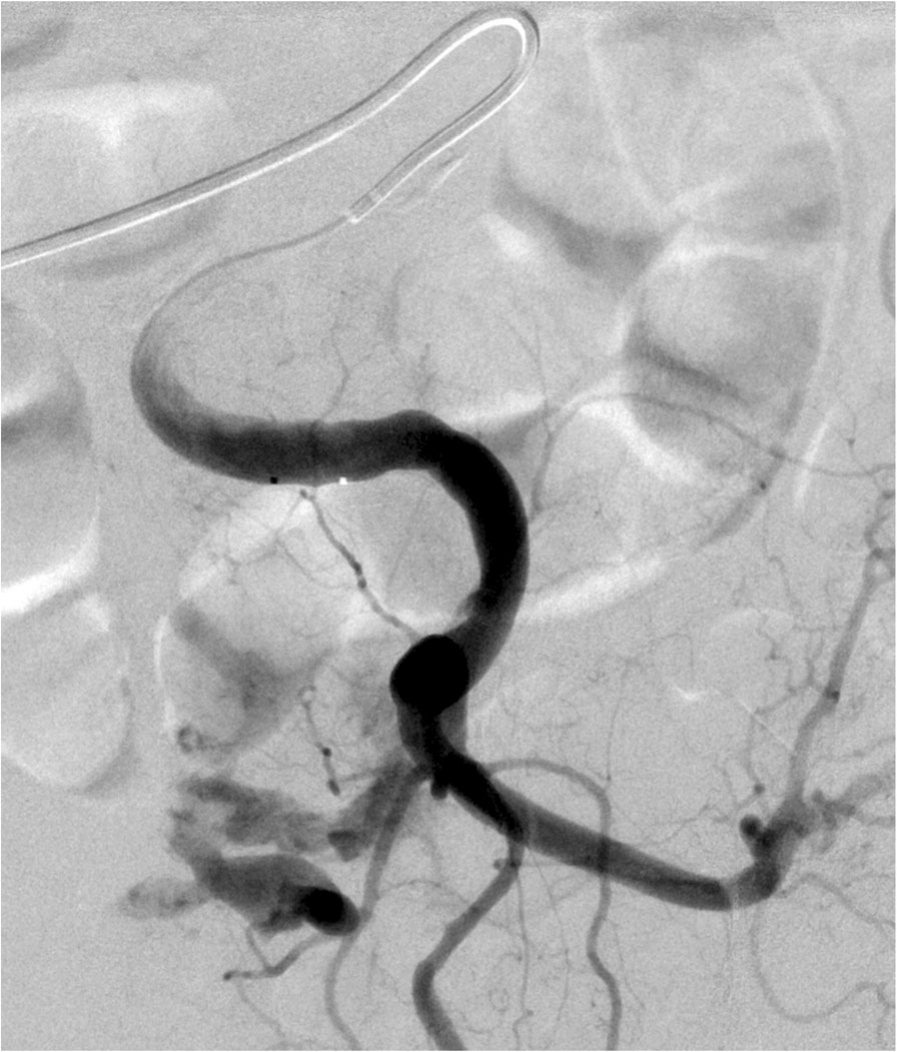
Catheterization of the left internal iliac artery with massive extravasation indicating active bleeding

An 86-year-old multiparous Japanese woman with an unremarkable medical history was transferred to our hospital after a traffic accident (Table 1). She was hit by a car while walking at a crosswalk. On presentation to our emergency department, she complained of pain in her buttock. Her Glasgow Coma Scale score was 15/15. Her blood pressure was 100/53 mmHg, heart rate was 93 beats/minute, respiratory rate was 15 breaths/minute, and oxygen saturation was 100% while breathing 2 L/minute of oxygen. A whole-body computed tomography scan revealed fractures of her left pubic bone and sacrum and a hematoma with contrast extravasation in front of the sacrum (Fig. 1). Her blood pressure then suddenly dropped to 67/38 mmHg secondary to hemorrhagic shock. Rapid resuscitation with fluids and blood was performed. We attempted to perform transcatheter arterial embolization (TAE). Based on angiographic findings (Fig. 2), bilateral internal iliac artery embolization was performed with gelatin sponge particles. She received 560 ml of packed red cells, 480 ml of fresh frozen plasma, and 200 ml of platelets, and she became hemodynamically stable.

**Table 1:** Laboratory findings on admission

Serum chemistry		
Na	141	mmol/l
K	4.1	mmol/l
Cl	102	mmol/l
Ca	9.5	mg/dl
Total protein	6.6	g/dl
Albumin	3.3	g/dl
Blood urea nitrogen	17.8	mg/dl
Creatinine	0.89	mg/dl
Total bilirubin	0.7	mg/dl
AST	43	IU/l
ALT	31	IU/l
LDH	449	IU/l
CK	217	IU/l
Blood cell count		
WBC	11.1 × 10^9^	/l
RBC	3.25 × 10^12^	/l
Ht	30.2	%
Hb	10.6	g/dl
Platelets	193 × 10^9^	/l
RBC fragmentation	0	
Coagulation tests		
PT-INR	1.1	
aPTT	35.1	seconds
FDP	1073	μg/dl

*ALT* alanine aminotransferase, *aPTT* activated partial thromboplastin time, *AST* aspartate aminotransferase, *Ca* calcium, *CK* creatine kinase, *Cl* chlorine, *FDP* fibrin degradation product, *Hb* hemoglobin, *Ht* hematocrit, *K* potassium, *LDH* lactate dehydrogenase, *Na* sodium, *PT-INR* prothrombin time-international normalized ratio, *RBC* red blood cell, *WBC* white blood cell

After admission to our intensive care unit, she developed shaking chills and a high fever. She was hemodynamically stable the following day. However, reddish urine was observed. Her serum lactate dehydrogenase level was extremely high, and fragmented red blood cells were present on peripheral blood smears. On the third day of hospitalization, despite fluid challenges and the use of diuretics, she became anuric and thus underwent hemodialysis. However, she developed severe delirium and was intubated under sedation. She was not diagnosed as having TTP at this point because her platelet count was not reduced despite the worsening of her hemolysis. Her prothrombin time and activated partial thromboplastin time were normal, and her fibrin degradation products were returning toward the normal concentration within 3 days of admission; therefore, disseminated intravascular coagulation (DIC) was excluded.

**Fig. 3:**
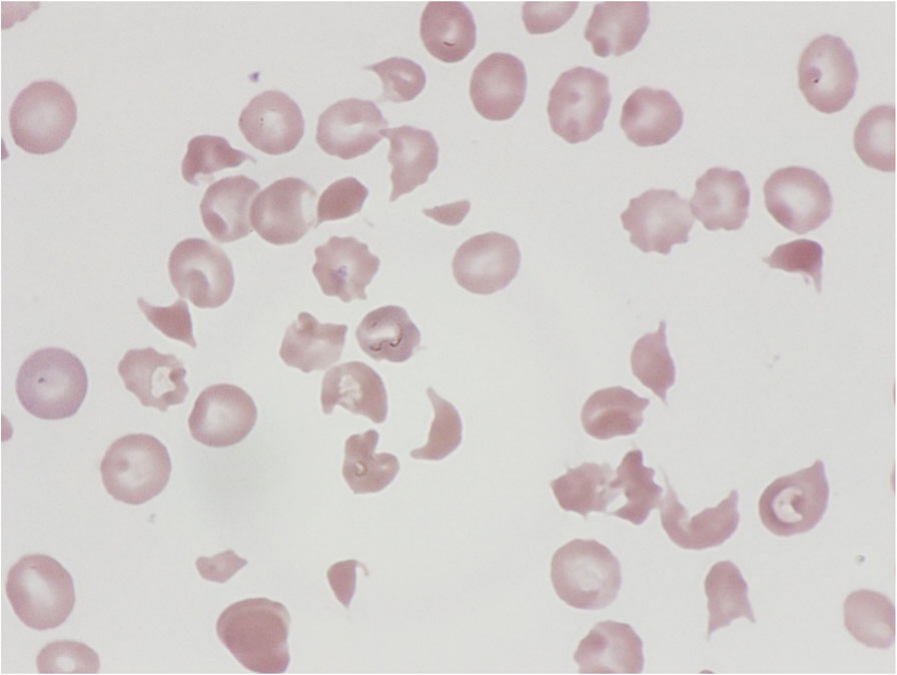
Peripheral blood smear shows multiple fragmented red cells. The platelet count is reduced

On the fifth day of hospitalization, her platelet count, measured by a different hemocytometer, was very low. Her fragmented red blood cell concentration measured by visual judgment based on the International Council for Standardization in Haematology (ICSH) reference method was 28.8% (Fig. 3). We finally confirmed the diagnosis of TTP based on the classic pentad of TTP and began plasma exchange.

**Fig. 4:**
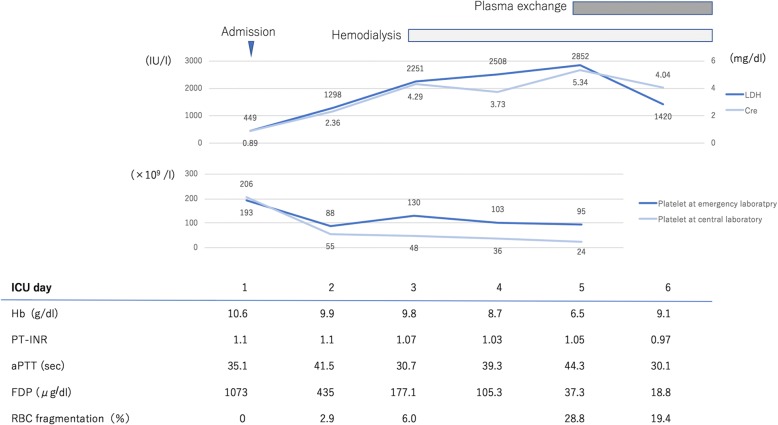
The clinical course of the patient. The serum lactate dehydrogenase level, creatinine level, fragmented red cells, and platelet count are shown. The platelet count reflects the fragmented red cells, which showed a spurious increase at the emergency laboratory. *aPTT* activated partial thromboplastin time, *CRE* creatinine, *FDP* fibrin degradation product, *Hb* hemoglobin, *ICU* intensive care unit, *LDH* lactate dehydrogenase, *PT-INR* prothrombin time-international normalized ratio, *RBC* red blood cell

After retrospectively checking her platelet count, we found that it had decreased to 55 × 10^9^/L on the second day of hospitalization (Fig. 4). The presence of many fragmented red cells is often associated with a spurious increase in the platelet count because the fragmented red cells are erroneously measured as platelets by automated blood cell counters.

Plasma exchange was continued for 5 consecutive days. Her clinical course dramatically improved in just a few days, and her platelet count increased. She was weaned from hemodialysis on the 15th day of hospitalization. She recovered fully and was discharged from our hospital on the 31st day of hospitalization. The ADAMTS13 activity measured by an enzyme immunoassay on the third hospital day was not reduced (65%), but a direct Coombs test was negative and the complement factor level was normal.

## Discussion

TMA, which includes TTP/hemolytic-uremic syndrome, is now a commonly used term. TMA is a pathological condition characterized by thrombocytopenia, microangiopathic hemolytic anemia, and organ injury. Primary TMA includes hereditary or acquired TTP, Shiga toxin-mediated hemolytic-uremic syndrome, complement-mediated TMA, drug-induced TMA, coagulation-mediated TMA, and metabolism-mediated TMA [1]. TTP was first reported by Moschcowitz in 1924 [2]. The onset of TTP appears to have a close association with ADAMTS13. TTP is typically defined as an ADAMTS13 activity of < 10% [3].

Secondary TTP may result from various conditions including connective tissue disease, transplantation, malignancy, pregnancy, and drugs [4, 5]. Surgery-induced TTP has also been reported in several cases [6], and it most commonly occurs after cardiac and vascular surgery [7]. The previously reported cases of secondary TTP did not necessarily rely on the detection of ADAMTS13 activity for the diagnosis. The mechanism of secondary TTP is less clear. In our patient, the ADAMTS13 activity was not significantly reduced, although she had the classic pentad of TTP. Post-traumatic TTP/TMA is quite rare. To the best of our knowledge, only one case report of TTP/TMA secondary to trauma has been reported in the English language literature [8]; this report was by Lim and Park [8], who described a case of TTP after blunt traumatic liver injury.

When thrombocytopenia occurs in association with trauma, we consider extravascular loss, consumption, dilution, DIC, and heparin-induced thrombocytopenia as differential diagnoses. Although DIC meets the pathological definition of TMA, here we describe DIC and TMA separately. The differential diagnosis of DIC and TMA is clinically difficult, but it is very important because the optimal therapy differs between the two conditions. Our patient rapidly developed thrombocytopenia and severe hemolysis despite normal coagulation test results, which was inconsistent with DIC. She did not have progressive anemia, and the hematoma did not increase in size on follow-up computed tomography. There was no possibility of hemolytic transfusion reaction, and we reaffirmed no problems based on the results of a cross-matching test. The development of hemolysis after intravascular treatment has been reported in cases involving closure of a patent duct arteriosus [9] and in association with infectious complications of *Clostridium perfringens* [10]. Our patient had severe vascular injury despite the presence of minor fractures; in addition, she received TAE. Whether the TMA was associated with the TAE in our case is unclear, but it is a possibility. Severe pelvic injury and embolization induce the endothelial cells of small vessels to produce high levels of unusually large von Willebrand factor multimers, which are higher than the level that can be processed by ADAMTS13.

Our patient’s clinical course dramatically improved due to plasma exchange in spite of the fact that administration of fresh frozen plasma was ineffective. This result may suggest that the effect of removing excess unusually large von Willebrand factor multimers, among several other effects of plasma exchange, contributed greatly to her outcome. Established guidelines advocate that treatment with plasma exchange should be initiated as soon as possible [11] if TTP is suspected. Plasma exchange might be the only possible life-saving therapy in patients with TMA following trauma.

In our hospital, the hematology analyzer (Celltac F; Nihon Kohden Corporation, Tokyo, Japan) at the emergency laboratory revealed little difference in the platelets versus the fragmented red cells, whereas that at the central laboratory (ADVIA 2120i; Siemens Healthineers, Erlangen, Germany) precisely analyzed them. The automated blood counter at the central laboratory used two-dimensional analysis through the addition of hemoglobin measurement, not just the size of the blood cells, allowing discrimination of platelets from fragmented red cells. When the platelet count is not consistent with severe hemolysis, we should reevaluate the thrombocytopenia with a different technique or visual counting method.

In conclusion, TMA must be considered in the differential diagnosis of unexplained thrombocytopenia and hemolytic anemia in patients with trauma. Plasma exchange must be performed without delay if TMA is suspected. Further progress in the clarification of TMA is expected in the future.

## Conclusions

It is important to recognize the possibility that TMA may occur after severe trauma. In the critical care setting, unexplained thrombocytopenia and hemolytic anemia should be investigated to eliminate the possibility of TMA.

## Abbreviations

DIC: Disseminated intravascular coagulation
TAE: Transcatheter arterial embolization
TMA: Thrombotic microangiopathy
TTP: Thrombotic thrombocytopenic purpura
